# Genome-wide evaluation of copy gain and loss variations in three Afghan sheep breeds

**DOI:** 10.1038/s41598-022-18571-4

**Published:** 2022-08-22

**Authors:** Mohammad Hossein Moradi, Roqiah Mahmodi, Amir Hossein Khaltabadi Farahani, Mohammad Osman Karimi

**Affiliations:** 1 Department of Animal Science, Faculty of Agriculture and Natural Resources, Arak University, Arak, 38156-8-8349 Iran; 2 Department of Animal Science, Faculty of Agriculture and Natural Resources, Herat University, Herat, Afghanistan

**Keywords:** Evolution, Genetics, Molecular biology

## Abstract

Copy number variation (CNV) is one of the main sources of variation between different individuals that has recently attracted much researcher interest as a major source for heritable variation in complex traits. The aim of this study was to identify CNVs in Afghan indigenous sheep consisting of three Arab, Baluchi, and Gadik breeds using genomic arrays containing 53,862 single nucleotide polymorphism (SNP) markers. Data were analyzed using the Hidden Markov Model (HMM) of PennCNV software. In this study, out of 45 sheep studied, 97.8% (44 animals) have shown CNVs. In total, 411 CNVs were observed for autosomal chromosomes and the entire sequence length of around 144 Mb was identified across the genome. The average number of CNVs per each sheep was 9.13. The identified CNVs for Arab, Baluchi, and Gadik breeds were 306, 62, and 43, respectively. After merging overlapped regions, a total of 376 copy number variation regions (CNVR) were identified, which are 286, 50, and 40 for Arab, Baluchi, and Gadik breeds, respectively. Bioinformatics analysis was performed to identify the genes and QTLs reported in these regions and the biochemical pathways involved by these genes. The results showed that many of these CNVRs overlapped with the genes or QTLs that are associated with various pathways such as immune system development, growth, reproduction, and environmental adaptions. Furthermore, to determine a genome-wide pattern of selection signatures in Afghan sheep breeds, the unbiased estimates of F_ST_ was calculated and the results indicated that 37 of the 376 CNVRs (~ 10%) have been also under selection signature, most of those overlapped with the genes influencing production, reproduction and immune system. Finally, the statistical methods used in this study was applied in an external dataset including 96 individuals of the Iranian sheep breed. The results indicated that 20 of the 114 CNVRs (18%) identified in Iranian sheep breed were also identified in our study, most of those overlapped with the genes influencing production, reproduction and immune system. Overall, this is the first attempts to develop the genomic map of loss and gain variation in the genome of Afghan indigenous sheep breeds, and may be important to shed some light on the genomic regions associated with some economically important traits in these breeds.

## Introduction

Livestock species play major socio-economic roles in the world, since they provide many goods and services to human populations. The sheep (*Ovis aries*) is one of the most important farm animal species, because of its high productive potential and its ability to adapt to harsh environments^1,2^. The breeding of sheep in Afghanistan dates back to about 9000 years ago when these valuable livestock species were domesticated^3^. The grassland in Afghanistan is around 30 million hectares, accounting for about 47% of Afghanistan's land area. The Hindu Kush slopes are a natural and permanent pasture for sheep breeding in Afghanistan and the production of sheep is of great importance in the livestock sector. Before the war and the recent drought, approximately 90% of the foreign exchange income of the livestock sector was derived from sheep, and livestock industry have a contribution up to 40% in country's exports^3^. Despite the importance of sheep production in Afghanistan, the lack of accurate pedigree and phenotypic records, are the main problems that limit the implementation of animal breeding programs.

Recently, the genome-wide association studies have successfully uncovered single-nucleotide polymorphisms (SNPs) associated with complex traits such as diseases and quantitative phenotypes^4^. However, various studies showed that these variations account for a small proportion of heritability^5^. With the development of high throughput techniques, abundant structural variations have been found in the genome, of which the main variations are copy number variations (CNVs). The CNVs are increasingly recognized as an important genetic source of different phenotypic traits^6–8^. CNVs have been shown to explain around 18% of genetic variation in the gene expression, having no overlap with that explained by SNPs^9^. Initial research on CNV traces to 1936, when C. Bridges found an association between the *BAR* “gene”, duplication of a part of a chromosome, and the eye size in Drosophila melanogaster^10^. Since 2006, several studies in humans and animals have identified the association between CNV polymorphisms and different complex phenotypic traits^7^. Several studies have shown the usefulness of CNVs data for describing the genetic basis of phenotypic variations in a variety of species like cattle^11,12^, dog^13^, chicken^14,15^, pig^16,17^, goat^18,19^, and rabbit^20^. As for sheep, Fontanesi et al.^21^ provided the first comparative map of CNVs in the sheep genome using a cross-species array comparative genome hybridization (aCGH). It has been reported that the duplication of the agouti signaling protein gene is associated with white coat color in sheep^22^, a gene duplication affecting expression of the ovine *ASIP* gene is responsible for white and black sheep^22^ and higher levels of *KIF2A* and *PHKG2* expression in Gansu Morden sheep are associated with disease resistance^23^. These studies suggest that identifying the CNVs distributed across the sheep genome can be very important for finding the genomic regions associated with some economically important traits in this species.

The aim of this study was to identify the genome-wide copy number variations (CNV) in three Afghan sheep breeds, and to study the associations between these regions of interest with different biological pathways. The results of this study, as one of the first attempts to develop the genome-wide map of CNVRs in local Afghan sheep breeds, can provide a valuable information genomic regions associated with economically important traits in these breeds.

## Materials and methods

### Animal samples

**Figure 1:**
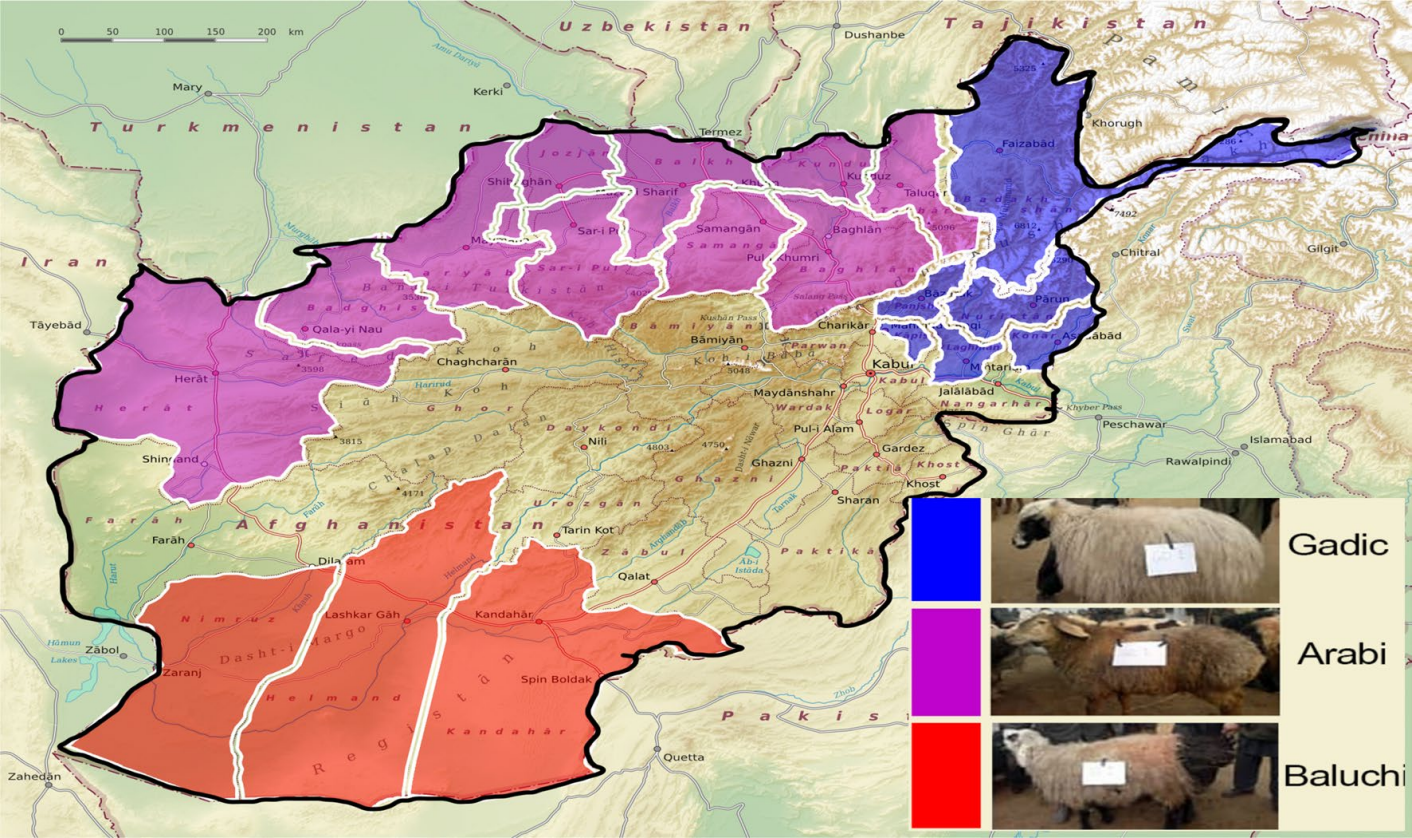
The studied breeds and their geographic distribution in different regions of Afghanistan (the figure has been created by using Adobe Photoshop CC 2020 v.21.0.2, https://www.adobe.com/products/photoshop/).

A total of 45 individuals, sampled from three breeds including 15 samples per each breeds of Arab, Baluchi and Gadik were collected for this study from different parts of Afghanistan (Fig. 1). The breeds used in this study are the most common and main indigenous sheep breeds reared in Afghanistan that have been prioritized over the past few years by animal breeding researches and private investment sectors^24^. Arab, Baluchi, and Gadik breeds account for around 13, 13, and 1% of total sheep reared in Afghanistan, respectively. Among these breeds, the Arabian sheep have a large body size and are the more capable for meat producing and known as a meat sheep, the Baluchi breed are well adapted to arid and desert environments and are known as dual purpose for wool and meat production and the Gadik breed as a wool sheep, is producing the best type of wool among other breeds^24^. The color of wools for each animal was collected at the time of blood sampling. While the color of wool for all Gadik sheep breed was white, a combination of different colors was observed in other two breeds specially in Baluchi. The white coats are favored in sheep for wool production that is easily dyed.

### DNA extraction, genotyping and quality control

The blood samples were collected via the jugular vein in the tubes containing EDTA (ethylene diamine tetraacetic acid) and the DNA was extracted using the GenElute Blood Genomic DNA Kit^24^. Genotyping was carried out in all animals using Illumina Ovine SNP 50K BeadChip, which contains 53,862 SNPs spanning the ovine genome. Quality control was implemented using the PLINK 1.90 software^25^. We removed the individuals and SNPs meeting any of the following criteria: individuals with a genotyping rate < 0.90; SNPs with > 0.05 missing data; and minor allele frequency (MAF) < 0.02^26^; SNPs with a P-value of < 0.000001 for the Fisher’s exact test of Hardy–Weinberg equilibrium (HWE). Finally, the SNPs without chromosomal or physical locations or located on the sex chromosome were excluded from final analysis. The markers located on the sex chromosomes were excluded from subsequent analyzes due to the different distribution of IBD (Identical by descent) pattern and the effective population size that affects identifying the CNVs^27^. This resulted in a data set of 45 individuals genotyped for 47,519, 46,430 and 47,711 autosomal SNPs in Arab, Baluchi and Gadik breeds, respectively.

### Identification of CNV and CNVR

CNVs were inferred within each individual using PennCNV v.1.0.5 software^28^. A Hidden Markov Model (HMM) algorithm as employed in PennCNV incorporates multiple parameters, such as signal intensity ratios (log R Ratio: LRR) and allelic frequencies (B allele frequency: BAF) values of each marker in each individual, and the population frequency of B allele (PFB) of SNPs. The software extracts the signal intensity of each allele from the array genotyping data and then combines allelic frequency information and single nucleotide polymorphism distance to identify CNVs. For this purpose, the values of X and Y, which is the signal intensity of alleles A and B were used. R is calculated as the total signal intensity of two alleles, or *R* = *X* + *Y*. As a normalized measure of total signal intensity, the LRR value for each SNP is then calculated as LRR = log2(R_observed_/R_Expected_), which R_observed_ is computed from linear interpolation of canonical genotype clusters^29^ obtained from a set of reference samples. The θ value measures the relative allelic intensity ratio of two alleles and is calculated from Eq. (1) which ranges from 0 to 1.

1 $$\theta = {\text{ arctan}}{\raise0.7ex\hbox{${\left( \frac{Y}{X} \right)}$} \!\mathord{\left/ {\vphantom {{\left( \frac{Y}{X} \right)} {\left( {\frac{\pi }{2}} \right)}}}\right.\kern-\nulldelimiterspace} \!\lower0.7ex\hbox{${\left( {\frac{\pi }{2}} \right)}$}}$$

The B Allele Frequency (BAF) refers to a normalized measure of relative signal intensity ratio of the B and A alleles^28^:

2 $${\text{BAF}} = \left\{ {\begin{array}{*{20}l} {0,} & {\quad if\; \theta < \theta_{AA} } \\ {{\raise0.7ex\hbox{${0.5 \left( {\theta - \theta_{AA} } \right)}$} \!\mathord{\left/ {\vphantom {{0.5 \left( {\theta - \theta_{AA} } \right)} {\left( {\theta_{AB} - \theta_{AA} } \right)}}}\right.\kern-\nulldelimiterspace} \!\lower0.7ex\hbox{${\left( {\theta_{AB} - \theta_{AA} } \right)}$}},} & {\quad if\; \theta_{AA} \le \theta \le \theta_{AB} } \\ {{\raise0.7ex\hbox{${0.5 + 0.5\left( {\theta - \theta_{AB} } \right)}$} \!\mathord{\left/ {\vphantom {{0.5 + 0.5\left( {\theta - \theta_{AB} } \right)} {\left( {\theta_{BB} - \theta_{AB} } \right)}}}\right.\kern-\nulldelimiterspace} \!\lower0.7ex\hbox{${\left( {\theta_{BB} - \theta_{AB} } \right)}$}},} & {\quad if\; \theta_{AB} \le \theta \le \theta_{BB} } \\ {1,} & {\quad if\; \theta \ge \theta \_BB } \\ \end{array} } \right\}$$

in which θ_AA_, θ_AB_ and θ_BB_ are the θ values for three canonical genotype clusters generated from external reference samples (such as HapMap samples). We retrieved signal intensity ratios (LRR) and allelic frequencies (BAF) for each SNP using the Illumina Genome Studio v1.0 software. To increase the confidence of the detected CNVs, quality control was performed by employing standard exclusions of the LRR (standard deviation of LRR) < 0.3, a BAF drift < 0.01 and a waviness factor < 0.05 (http://www.openbioinformatics.org).

CNV regions (CNVR) were determined by aggregating overlapped CNVs, identified in different animals, as reported previously^30^. For construction of the CNVR map, we classified the status of these CNVR into three categories, ‘Loss’ (CNVR containing deletion), ‘Gain’ (CNVR containing duplication) and ‘Both’ (CNVR containing both deletion and duplication). Chromosomal distribution of CNVRs in Afghan sheep breeds has been developed in R v4.0.2. The VENN diagram web tool was also used to create a Venn diagram showing the overlap between CNVRs identified in different breeds [https://bioinformatics.psb.ugent.be/webtools/Venn/ (accessed on July 3, 2021)].

Finally, to evaluate the CNVRs identified in the present study in more detail, we compared them to the CNVRs identified in an Iranian sheep breed. In particular, 96 animals belong to Iranian Baluchi sheep breed were used. This breed is rearing in eastern parts of Iran, close to the borders of Afghanistan, and genotyped using the Illumina Ovine SNP50k Beadchip that fully described previously by Gholizadeh et al.^31^. The same statistical methods used in this study was applied to detect CNV and CNVRs in Iranian Baluchi sheep.

### Genome-wide detection of selection signatures

To determine a genome-wide pattern of selection signatures in Afghan sheep breeds, the unbiased estimates of F_ST_ (Theta) was calculated as described by Weir and Cockerham^32^. Three comparisons consisting Arabi vs Baluchi, Arabi vs Gadik and finally Baluchi vs Gadik was performed. The values can theoretically range from 0 (showing no differentiation) to 1 (indicating complete differentiation, i.e. populations are fixed for different alleles). For each set of 5 adjacent SNPs, the average of F_ST_ values was calculated and termed windowed F_ST_. This is an approximate method of looking for regions where selection is apparent over multiple markers, rather than one-off high values. A window of 5 markers was chosen as it appeared to provide the better signal compare to other arbitrary window sizes^26^. The windowed F_ST_ values were then plotted against genome location. All scripts were written and performed in R v 4.0.2 (https://cloud.r-project.org/).

### Gene and QTL content and Cytoscape analysis

Genes harbored in the previously identified CNVRs were obtained from the Ensembl Genes 64 Database using BioMart software (https://www.ensembl.org/biomart/) based on the *Ovis* *aries* gene sequence assembly (Oar_v3.1). To functionally annotate these CNVRs, the gene ontology analysis (GO)^29^ was conducted using the Database for Annotation, Visualization, and Integrated Discovery Cytoscape functional annotation tool (http://www.cytoscape.org/). Also, to better understand the functions of the genes within the detected CNVRs, the *O. aries* Ensembl gene IDs were converted to human ortholog Ensembl gene IDs using BioMart because annotation of the sheep genome was limited^33^. In addition, to study whether these CNVRs were associated with economically important traits, these CNVRs were compared with QTLs in the sheep QTL database (https://www.animalgenome.org/cgi-bin/QTLdb/OA/index, sheep QTL database, accessed on 10 May 2022).

### Approval for animal experiments

The experimental protocols were approved by the Biomedical Ethics Committee of Arak University. All methods were carried out in accordance with relevant guidelines and regulations. The authors also complied with the ARRIVE (Animal Research: Reporting of In Vivo Experiments) guidelines.

## Results and discussion

### Genome-wide detection of CNVs

The large-scale CNV explorations was performed and the results identified 411 CNVs across 45 individuals from three breeds, where the total length was 144 Mb and the average length for each individual was 9.13 Mb (Table 1). Among them 306, 62 and 43 CNVs was observed in Arabian, Baluchi and Gadik sheep breeds, respectively. The length of CNVs compared to whole-genome length, per each animal was 1.13%. The average length of the CNVs was 352 kb, and the median length was 214 kb. We found that approximately 24% of the CNVs range from 20 to 100 kb, 55% of CNVs range from 100 to 500 kb in size, and 21% being larger fragment CNVs (> 500 kb) (Table 1).

**Table 1:** Summary of CNVs identified in Afghan sheep breeds.

Descriptive statistics	Arab	Baluchi	Gadik	All animals
Number of CNVs	306	62	43	411
Total length of CNV (kb)	128,897	9864	5872	144,635
The proportion of CNVs length divided by whole-genome length per each animal^1^	3.44	0.26	0.17	1.31
The average length of CNVs (kb)	421	159	137	352
The median length of CNVs (kb)	301	103	102	214
Number (percentage) of CNVs range 20–100 kb	51 (17%)	28 (45%)	21 (49%)	100 (24%)
Number (percentage) of CNVs range 100–500 kb	173 (56%)	32 (52%)	22 (51%)	227 (55%)
Number (percentage) CNVs with the length of > 500 kb	82 (27%)	2 (3%)	0	84 (21%)

^1^The length of the sheep genome is considered to be 2500,000 kb (https://www.ensemble.org).

In this study, CNVs were observed in all autosomal chromosomes, that was in agreement with the results obtained in previous studies in Chinese sheep^34^, native Korean Hanwoo cattle^35^, Chicken^14^ and human^36^. The average of CNVs per each animal in the present study was 9.13. A variety of CNVs number per each individual have been reported, including less than 1^23^ to 16.9^21^ in sheep, 17.9 in goat^18^, 9.6 in chicken^14^, 3.1 for horse^37^, and 19.16 in Iranian native cattle^38^. The number and the length of CNVs are influenced by a variety of factors including the sample size, the statistical method, species, and markers density^39,40^. For instance, while 45 samples from three Afghan sheep breeds (15 samples per each breed) have been used in the present study, Fontanesi et al.^21^ studied 11 samples (1 up to 2 samples per each breeds) to develop the first comparative map of CNVs in Italian dairy or dual-purpose sheep breeds using a cross-species array comparative genome hybridization (aCGH). Ma et al.^23^ were identified the CNVs in the genomes of eight sheep breeds (20 samples per each breed) using the sheep SNP50k BeadChip genotyping array, where, a total of 173 CNVs were discovered by the average and median sizes of 123 kb and 100 kb, respectively. The number of CNVs obtained in Afghan sheep breeds were in the range of previously reported studies by Fontanesi et al.^21^ and Ma et al.^23^ and the differences may be caused due to different breeds of animal and the number of samples.

### Genome-wide identification of autosomal CNVRs

CNVRs were determined by merging the overlapping CNVs identified in all samples, as reported previously^6,30^. A total of 376 autosomal CNVRs (125 losses, 242 gains, and 7 both event) were identified. The average and median sizes of CNVRs were 361 kb and 224 kb, respectively. Approximately 25% of the CNVRs ranged from 20 to 100 kb, 53% ranged from 100 to 500 kb and 22% of the CNVRs were larger than 500 kb (Table 2). Detailed information on CNVRs in Afghan sheep breeds is presented in Table 2.

**Table 2:** Summary of CNVRs identified in Afghan sheep breeds.

Descriptive statistics	Arab	Baluchi	Gadik	All
Number of CNVRs in deletion state	50	44	31	125
Number of CNVRs in duplication state	233	3	8	242
Number of CNVRs in deletion/duplication state	3	3	1	7
Total length of CNVR (kb)	122,811	7667	5275	135,756
Average of CNVRs length (kb)	429	153	132	361
Median of CNVRs length (kb)	306	100	100	224
Number (percentage) of CNVRs range 20–100 kb	46 (16%)	27 (54%)	20 (50%)	93 (25%)
Number (percentage) of CNVRs range 100–500 kb	160 (56%)	21 (42%)	20 (50%)	201 (53%)
Number (percentage) CNVRs with the length of > 500 kb	80 (28%)	2 (4%)	0	82 (22%)

**Figure 2:**
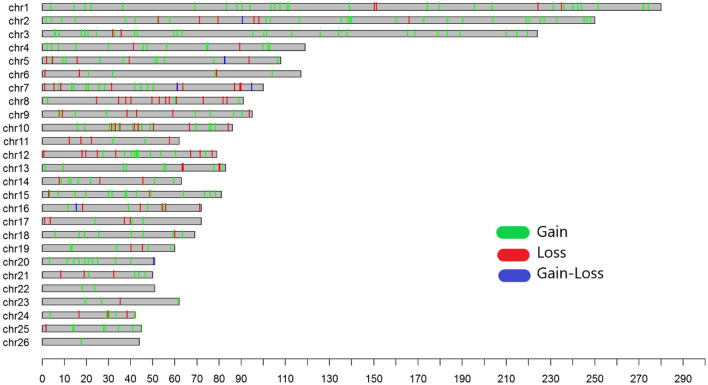
Chromosomal distribution of copy number variant regions (CNVRs) in Afghan sheep breeds, developed using R v4.0.2 (https://cloud.r-project.org/): in this figure the chromosome length (Mb) is displayed on the X-axis and CNVR variations with deletion, duplication, and deletion/duplication state showed on different chromosomes on Y-axis.

The map of the CNVRs distribution on autosomal chromosomes was created in total samples of Afghan sheep and has been shown in Fig. 2.

Keeping in mind that the detection rate of CNVRs is affected by many factors^41^, the results of this study were compared with previous studies in sheep. Between 111^42^ and 3488^43^ CNVRs have been detected in previous studies, with CNVR lengths of 10.56^21^ up to 120.53^23^ Mbs. The CNVRs identified in these studies were different to some extent, which may be due to the differences in sheep breeds, CNV detection platforms and CNV calling algorithms used. Similar findings were also observed in CNVR studies conducted in different mammals including pig^44^ dairy and beef cattle^45^ and goat breeds^46^. The use of different CNV calling algorithms has a substantial effect on the results of CNVR studies. Currently, three algorithms for CNV detection based on SNP arrays have been developed, which are available in different programs, including PennCNV, cnvPartition and QuantiSNP^23^. Compared to other algorithms, PennCNV provides a standard proven method that is more reliable for assessing the number of copies when using Illumina arrays because it incorporates the allelic intensity ratio at each SNP marker and the total signal intensity, the allele frequency of SNPs, the distance between neighboring SNPs, and the GC content to overcome biases^47^. Also, the software packages currently commonly used for CNV detection include PennCNV, CNVcaller, and CNVnator, each of them have their own advantages and disadvantages. PennCNV software has been extensively applied to Illumina chip data^48^ while, CNVcaller and CNVnator software are using read depth methods to detect CNVs in resequencing data^33,49^.

The results indicate that the number of CNVRs in the chromosomes varies from 1 to 39 (Additional file Table S1), with the top three highest rate of CNVRs (more than 30) located on chromosomes 2, 1, and 3, which contained 39, 34, and 31 CNVRs respectively. Chromosome 26 had the smallest number of CNVRs. This observation was in agreement with Yuan et al.^50^ where they reported that the chromosome length had a significant positive linear relationship with the number of CNVRs (R^2^ = 0.87) with the maximum found in chromosome 1 and the minimum found on 26. Conflicting results have also reported by Jenkins et al.^43^ in Texel, Coopworth, Perendale, Romania, and Merino sheep and Zhu et al.^48^, in the study of CNVR-containing genomic regions in Tibetan sheep, stated that chromosome 3 had the highest number of CNVRs. These results may be influenced by the number of samples and arrays used. For example, while 50k chips were used in the present study, Zhu et al.^48^ used the chips with a 600k marker density. However, the medium density of Illumina OvineSNP50 BeadChip array used in our study, provides a standard proven method to detect CNVs and has been widely used in CNV detection in different sheep breeds^41,51–53^. It was also observed in the present study that CNV duplication state is more than the deletion state, which may be because the deletions are under stronger purifying selection (which removes deleterious variants from the population) than duplications, therefore deletions should on average, be both less frequent and smaller than duplications^30^.

**Figure 3:**
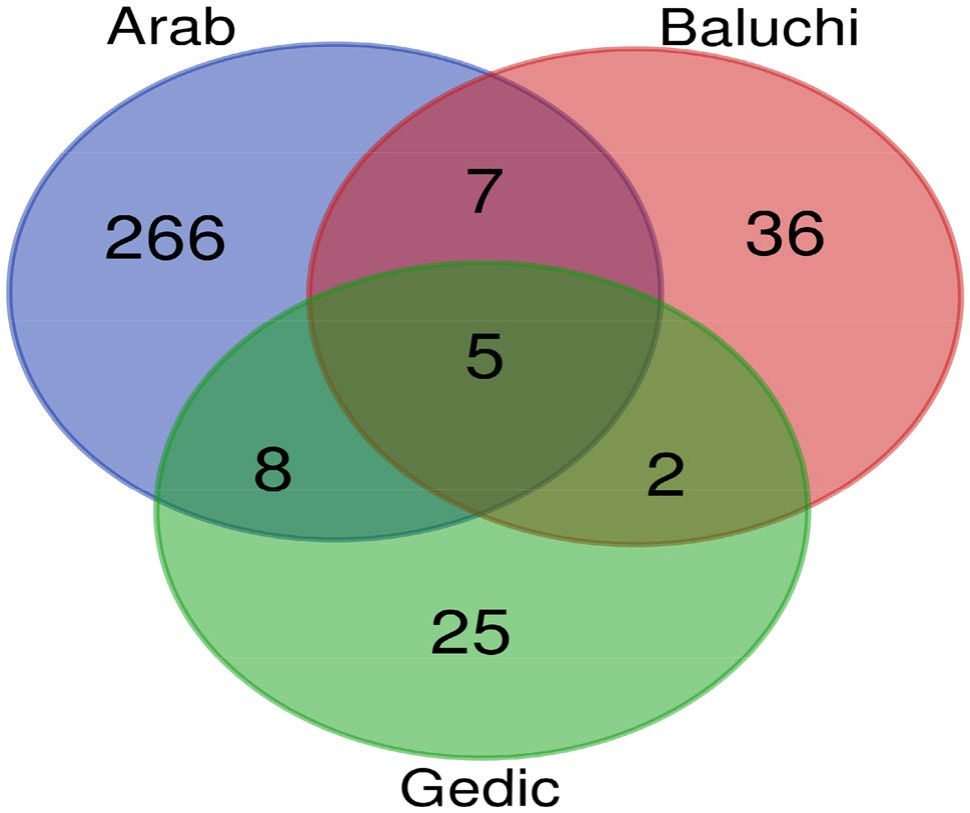
Venn diagram representing common and unique CNVRs found among the three Afghan sheep breeds. This figure was created using VENN diagram web tool (https://bioinformatics.psb.ugent.be/webtools/Venn/).

A comparison of the CNVRs among breeds is shown in the Venn diagram (Fig. 3). The highest number of shared CNVRs was observed between Arabi and Gadik (n = 8), and the lowest number, was shared between Baluchi and Gadik (n = 2). Long-term adaptation to different environmental conditions or different selection schemes increases the variation in CNVRs among breeds^30^. Similar environmental conditions, gene flow, and shared ancestral components may have led to an increase in shared CNVRs among breeds^41^. The more CNVRs identified in Arab sheep and the highest shared CNVRs observed between Arab and Gadik are in agreement with the distribution of these breeds in Afghanistan. Arab sheep breed is rearing in a wider geographic ranges and therefore, is adapted to different climatic conditions. Also, Arab and Gadik sheep breeds are rearing close to each other and gene flow may have occurred during last generations, leading to an increase in shared CNVRs among breeds (Fig. 1).

### Gene content and functional annotation

The BioMart system in ENSEMBL was used to retrieve the gene content in the 376 CNVRs (125 losses, 242 gains, and 7 both event). Our results revealed that some CNVRs did not overlap with any annotated gene that was in agreement with the results reported in previous studies on cattle^54^ and sheep^52^. This may be due to the genome assembly version, especially in non-human species^54^, and also several studies have highlighted the genome-wide distribution of CNVs in regions covering non-coding sequences, thus affecting the regulation of distant target genes^55^. However, many genes identified in these regions had economically important biological functions including *IL37* genes, affecting immune response, *DCT* gene with an important role in melanin synthesis and color expression in skin and feathers, *CACNA1A* gene influencing contraction process muscles and the *TCF12* gene affecting skeletal muscle, thyroid, B and T cells (GeneCards). The *IL37* gene is located on chromosome 3, which is involved in pro-inflammatory and anti-inflammatory reactions that it is one of the candidate genes influencing the immune response and resistance to mastitis in cattle^56^. The *DCT* gene is located on chromosome 10, which is involved in the production of the pigment Eomelanin and Feomelanin. In mammals, skin and hair color are depending on the relative ratio of Eomelanin and Feomelanin^52^. The *CACNA1A* gene on chromosome 5 involve in muscle contraction, hormone or neurotransmitter secretion, and gene expression (GeneCards). Zhu et al.^57^ have shown that this gene is associated with fat deposition in fat tailed sheep breeds. The *TCF12* gene which is situated on chromosome 7, is expressed in many tissues, including skeletal muscle, thyroid, B, and T cells (GeneCards). Dunner et al.^58^ reported that this gene affects muscle fatty acid production in Bos Taurus cattle.

To determine the underlying biological functions of the genes overlapped with CNVRs, the Gene Ontology (GO) analysis was used and the genes were evaluated for their affects on biological process (BP), cellular component (CC), molecular function (MF), and immune system. Our results showed that the most of the genes were associated with sensory perception and immune system activity, interleukins, and growth performance (Table 3). The same pathways have previously been reported for the development of skeletal muscle and immune system in Altai and Tibetan sheep^48^ and in antigenic processes and immune responses by Fontanesi et al.^21^ and Hou et al.^34^. Liu et al.^6^ reported the genes that contributed in the adaptation to environmental conditions, which is consistent with the results of the present study.

**Table 3:** Summary of the results from the ontology of genes identified in CNV regions in all samples.

GOID	GO term	P-value
GO:0007600	Sensory perception	1.9E−6
GO:0050907	Detection of chemical stimulus involved in sensory perception	9.5E−5
GO:0050906	Detection of stimulus involved in sensory perception	1.4E−11
GO:0007606	Sensory perception of chemical stimulus	1.8E−11
GO:0007608	Sensory perception of smell	1.4E−13
GO:0048588	Developmental cell growth	9.8E−3
GO:0005149	Interleukin-1 receptor binding	4.5E−5

**Figure 4:**
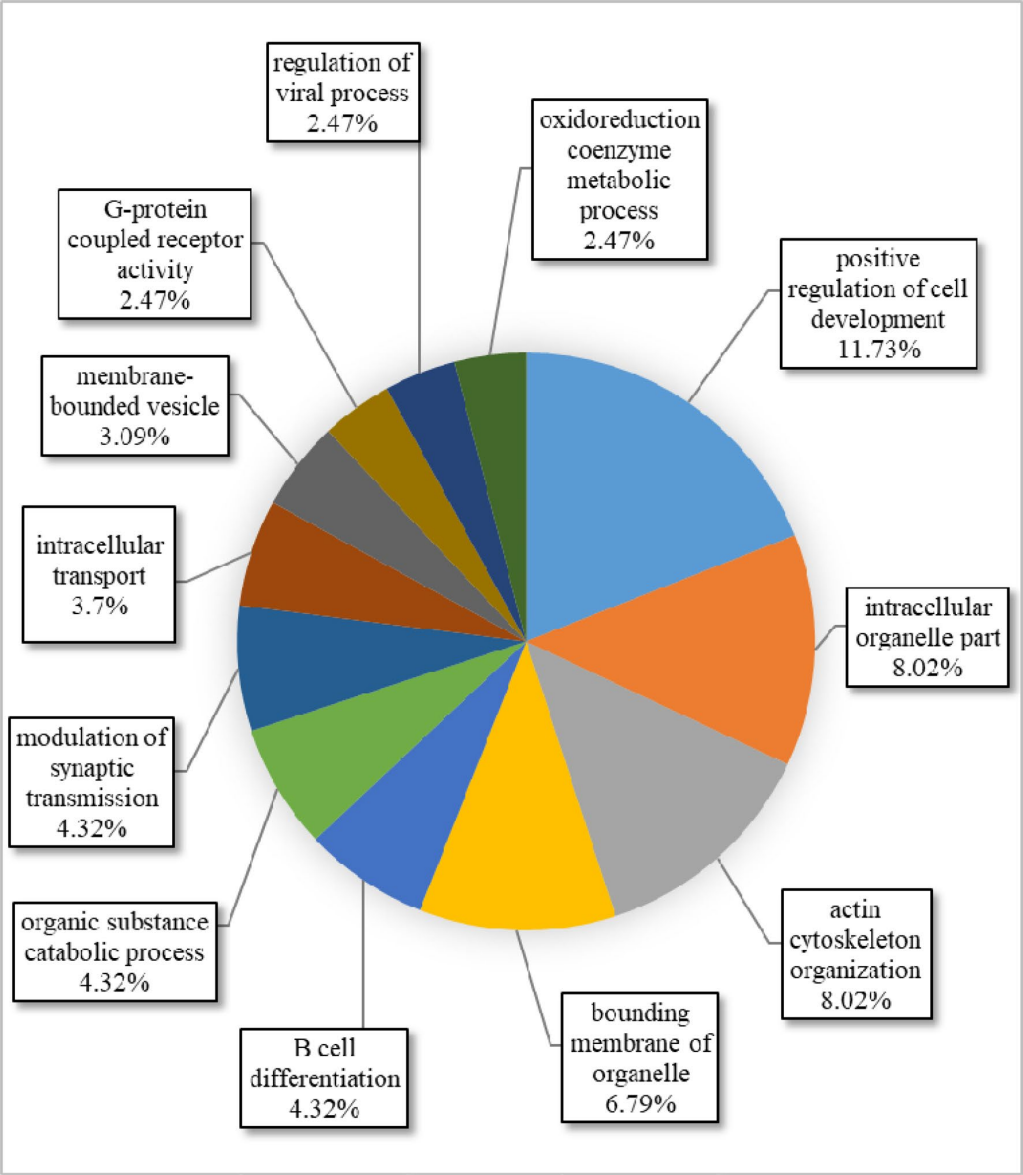
Circular diagram of the biological pathways involved by the genes reported in CNV-containing regions of all Afghan sheep breeds produced by Cytoscape functional annotation tool (http://www.cytoscape.org/): in this figure, the name of each process and the degree of the gene participation have been reported. The pathways with a participation rate of less than 2% are not shown.

A circular diagram of the biological pathways involved by the genes reported in CNVR-containing regions of all Afghan sheep breeds has been shown in Fig. 4. The results showed that 11.73% of the genes affected the positive regulation of cell development. This pathway refers to any process that increases the speed, frequency, or rate of development of a cell from its formation into a mature structure (QuickGO). The ratios of 8.02, 8.02, and 6.79%, were also influencing the pathways of intracellular organelle part, actin cytoskeleton organization, and binding membrane of organelles, respectively (Fig. 4). The identification of GOTerms in this study and their comparisons with other studies showed a number of similar pathways. Most of these similar pathways include the G-Protein coupled receptor signaling pathway (GO:0007186)^6,34^, Sensory perception (GO:0007600)^6^, neurological system process (GO:0050877)^34^, Cation binding (GO:0043169)^7^, metal ion binding (GO:0046872)^7^ and enzyme binding (GO:0019899)^23^. The study of these GOTerms in other species also showed some reported pathways that are consistent with the present study, for instance, G-Protein coupled receptor signaling pathway (GO:0007186) in cattle^59^.

**Figure 5:**
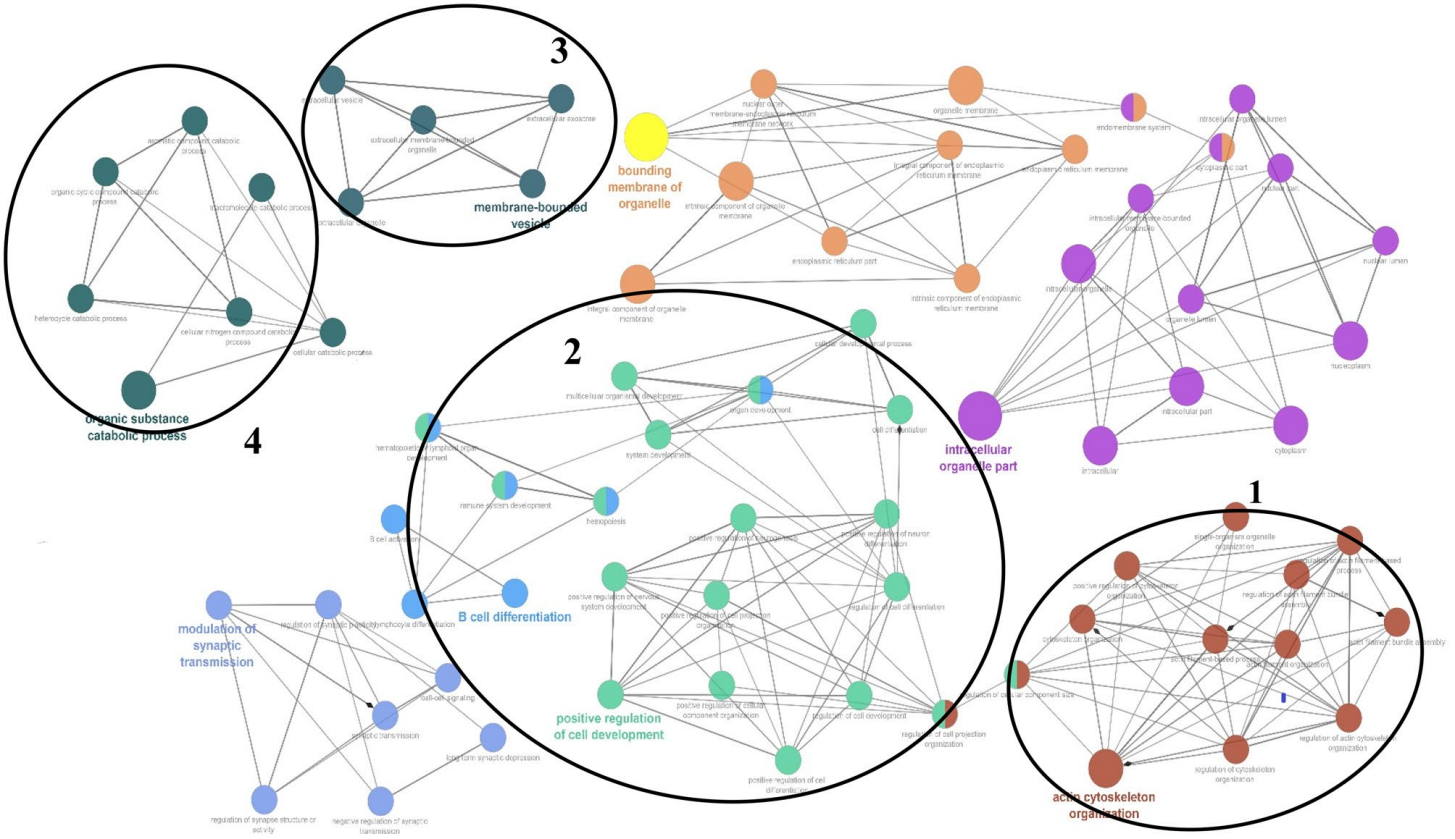
Gene-network interactions between all the genes reported in the regions containing CNVRs in Afghan sheep breeds, produced by Cytoscape functional annotation tool (http://www.cytoscape.org/). Circles of different colors represent (biochemical pathways) and the lines between these circles indicate the interactions between these pathways with each other. Zone 1 include the genes that affecting the skeletal muscle, zone 2 influence the birth weight, fertility, embryonic development and skeletal morphology, zone 3 is containing the genes that effect on skeletal development, cartilage structure, and joint function and zone 4 plays a role in fetal development and development.

The gene networks involved by the genes identified in the present study are shown in Fig. 5. Four gene networks related to different traits were created and several co-associated genes in these networks are involved in biological processes previously found to be associated with production and reproduction performance traits. Network 1 in stance, included different genes, such as *ZNF* and *CAPZB* that were previously reported to be associated with muscle structure and meat production^60^. Genes in cluster 2 primarily included *HINT2*, *TNFRSF19*, *CCIN*, *FNDC3A*, *SYCP2*, *TUBGCP3*, and *RECK*, which were related with birth weight, body size, fertility, embryonic development, as well as skeletal morphology^61,62^. Cluster 3 included some genes such as *ACAN* and cluster 4 the genes like *NID2* which were previously reported to be associated with skeletal development and cartilage structure^36^ and embryonic development^63^, respectively.

**Figure 6:**
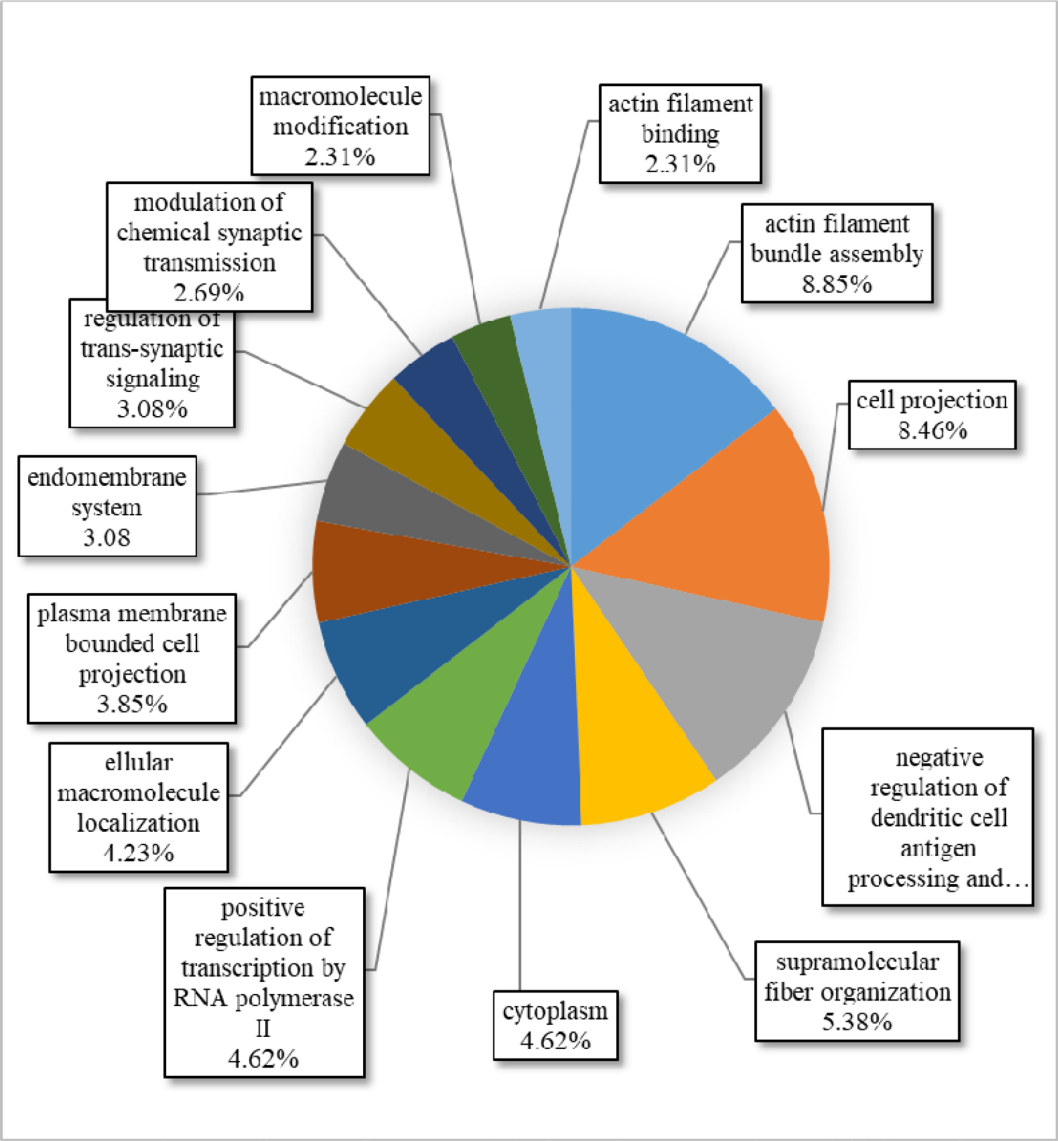
Circular diagram of the biological pathways involved by the genes reported in orthologous regions containing CNVs in *H. sapiens*, created by Cytoscape functional annotation tool (http://www.cytoscape.org/): In this figure the name of each process and its percentage has been showed. Pathways with a contribution of less than 2% were removed from the graph.

As the current annotation of the sheep genome is not as comprehensive as *H. sapience*^26^, the regions of interest in *O. aries* were compared to the corresponding areas in human (Fig. 6). The results of gene ontology showed that 8.85% of the genes that overlapped with CNV regions affected the actin filament bundle assembly pathway. This pathway consists a set of actin filament bands that are aligned in an axis with identical or opposite poles (approximately 30–60 nm apart) (QuickGO). Also, the ratios of 8.46, 7.31, and 5.38% were associated with cell projection, negative regulation of dendritic cell antigen presentation, and supramolecular fiber organization, respectively (Fig. 6). Given that actin and myosin are one of the most important components of muscle development, it seems that the development of the skeletal and muscle systems has been one of the most important targets during the evolution of the Afghan sheep. These pathways had a P-value of 3.4E−7, 3.2E−5 and 1.2E−11 in *O. aries* and 2.6E−3, 4.5E−5 and 6.6E−9 in *H. sapience*, respectively.

**Figure 7:**
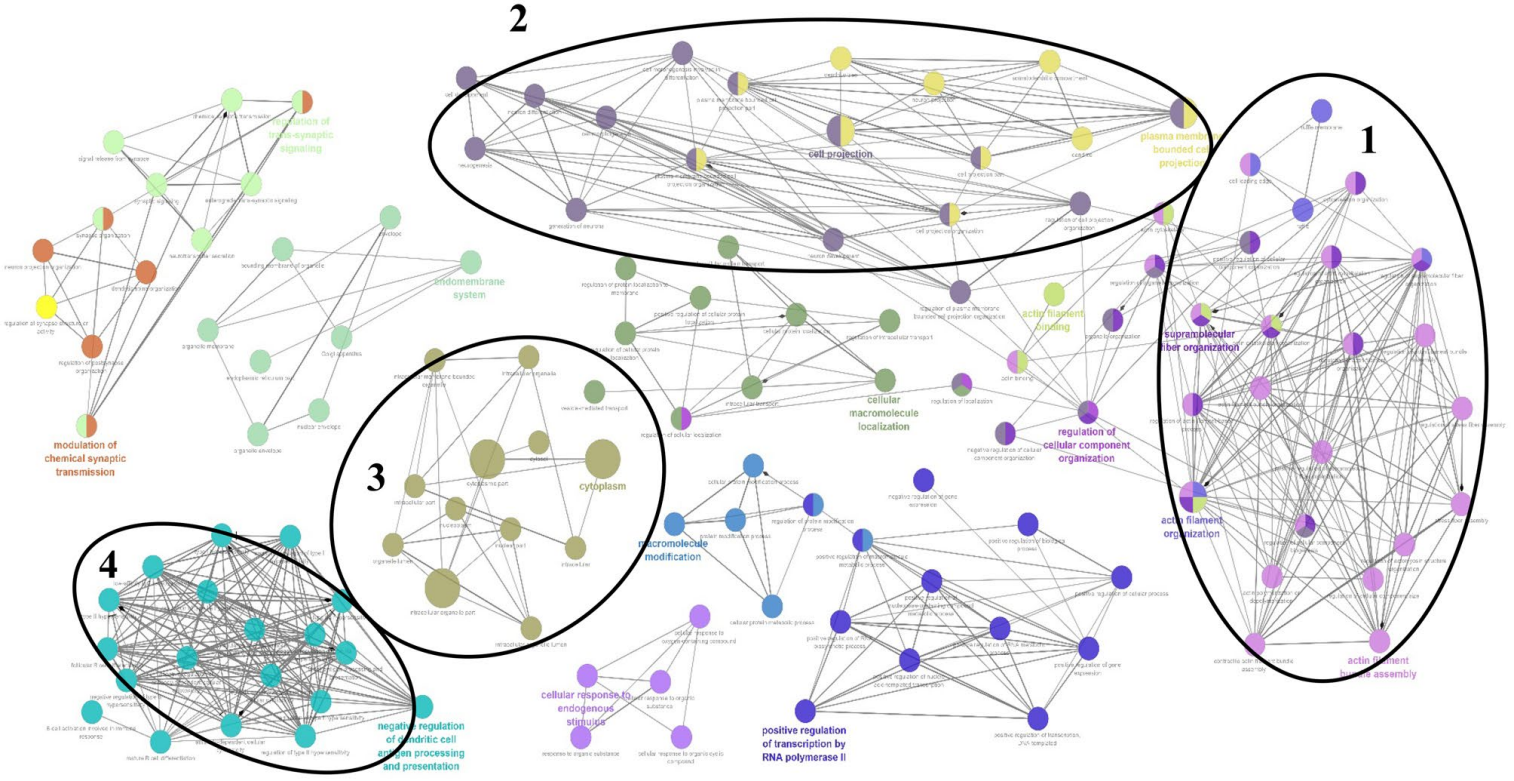
Gene-network interactions between all the genes reported in the orthologous areas of *H. sapience*, for the genomic regions containing CNVRs in Afghan sheep breeds [the figure developed by Cytoscape functional annotation tool (http://www.cytoscape.org/)]. Circles of different colors represent biochemical paths and the lines between these circles indicate the interactions between these paths with each other, whose intensity is a measure of the assurance of a functional partnership. Region 1 contains the genes affecting the muscle skeletal region, region 2, the sex determination of males and females, birth weight, carcass weight, fetal development, skeletal development and cartilage structure region 3 is associated with birth weight and skeletal morphology and body size, and finally, region 4, affect the immune response, antigens, and antibodies.

Also, the gene networks developed by the genes identified in orthologous regions of *H. sapience* were also evaluated and the results revealed that some networks are associated with muscle skeletal, embryonic, reproductive stages, birth weight, melanocyte, pigmentation, enteric nerves, skeletal development, immune response, antigens and antibodies (Fig. 7). Network 1 in stance, included different genes, such as *ROCK-1*, *ROCK-2* that were previously reported to be associated with muscle structure system in sheep^64^ and network 2 included *PTGDR*, *HINT2*, *SPAG8*, *CCIN*, *TNFRSF19* and *ACAN* genes which were related with sex determination^65^, fertility, fetal development, birth and carcass weights^66^ and skeletal development and cartilage structure^36^. Cluster 3 included some genes such as *SYCP2, NPR2, EDN3* influencing birth weight, skeletal morphology and body size^67^, and finally network 4 including the genes like *PRSS55, CRISP2* that are relate with immune system, antigens and antibodies^68^.

Due to the high conservation of the genes between humans and sheep^69^, genes that are known to be related to complex human traits may also be important for related traits in sheep. However, further research will be needed to formally test the functional relevance of these genes.

### QTLs overlapping with identified CNVRs

To further reveal the CNVRs associated with sheep economic traits, the detected CNVRs were compared with quantitative trait loci (QTLs) in the sheep QTL database. We found 121 CNVRs overlapping with 171 QTLs, including milk production and quality, growth and carcass traits, reproduction performance, fleece yield, fecal egg counts, tail fat deposition, horn type and health-related traits (Additional file Table S1).

Many studies have shown that CNVRs contain QTLs associated with important economic traits in animals^50,70^. Therefore, the CNVRs detected in this study were compared with the QTLs reported in the sheep QTL database. The QTL categories found in this study were basically identical to those found in previously reported studies^50,52,71^. For example, the health-related QTLs found included fecal egg count QTLs, and worm count QTLs that previous studies have reported that worm disease infection rates in sheep can exceed 70% in many countries, causing huge losses to the livestock industry^72^. Relative to barn-fed livestock, grazing livestock are more likely to be infected with worms. These results indicate that CNVs, which are a critical type of genetic variation, may have an important effect on sheep health.

### Comparison of identified CNVRs with the regions that have been under selection signatures

**Figure 8:**
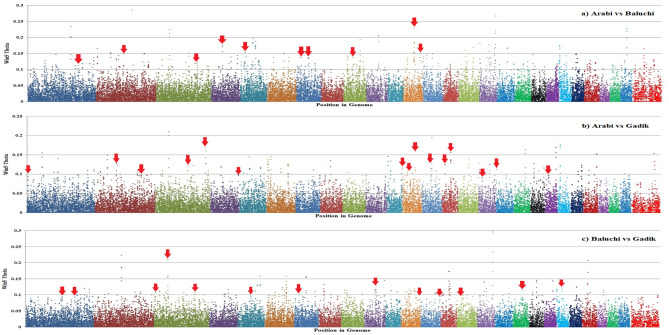
Distribution of selective sweep signatures for different comparisons in Afghan sheep breeds by chromosome. SNP position in the genome is shown on the X-axis and each chromosome was highlighted in different colors, and windowed F_ST_ (Theta) is plotted on the Y-axis. The red arrows are highlighting the peaks that overlapped by CNV regions identified in our study. This figure was created using R v 4.0.2 (https://cloud.r-project.org/).

To identify loci that have been targets of selection across different Afghan sheep breeds, the windowed F_ST_ was plotted against genomic location (Fig. 8). Variants with unusually large F_ST_ values are typically interpreted as being the targets of local selective pressures due to the hitch-hiking effect^26^.

Comparison of identified CNVRs with the regions that have been under selection signatures indicated that, 37 of the 376 CNVRs (~ 10%) were also identified to be under selection pressure and confirmed by selective sweep signatures (Fig. 8 and Additional file Table S1). Almost all of these regions overlapped with the genes or QTLs that had previously been identified to be associated with production, reproduction and environmental adaptions traits in sheep (Additional file Table S1, Sheet 2). Zhu et al.^57^ identified the CNVs and selection signatures on the X chromosome in Chinese indigenous sheep breeds with different tail types. Their results revealed 20 common genes that were shared by CNVRs and selection signatures, and were associated with fat, energy metabolism, and immune system.

### Comparison of identified CNVRs with Iranian sheep breed

Finally, to characterize the CNVRs identified in the present study in more detail, we compared them to the CNVRs identified in an Iranian sheep breed namely Baluchi. This breed is fat-tailed and dual-purpose breed (wooly and meaty), which is well adapted to the dry and hot climate conditions of eastern Iran. Iranian Baluchi sheep has the largest population than other breeds of sheep in Iran (about 30% sheep population) and have selected for this comparison since, it is mostly rearing in the regions of Iran that are close to the boarder of Afghanistan^31^. After quality control, 45,673 autosomal SNPs from 87 animals were used for CNVRs detection. The results indicated that 20 of the 114 CNVRs (18%) identified in Iranian sheep breed were overlapped or located close to the CNVRs identified in our study (Table 4). Study of the genes reported in these regions of interest revealed that most of those overlapped with the genes influencing production, reproduction and immune system (Table 4).

**Table 4:** The list of CNVs overlapped between Afghan and Iranian sheep breeds and the genes and their functions reported in these locations.

CNVs positions in Afghan sheep	CNVs positions in Iranian sheep	Distance (kb)	The genes reported in this location	Function of the gene	References
Chr1:242324429–242574074	Chr1:241583858–241653162	671	*SLITRK3*	Milk production traits	^73^
Chr2:172582236–172616581	Chr2:172272878–172424726	158	*MBD5* and *EPC2*	Growth traits	^74^
Chr5:39479796–39659262	Chr5:39400849–39585301	0	*COL23A1*	Fertility	^75^
Chr6:78371034–79041954	Chr6:78371034–78600405	0	*EXOC1L*	Reproduction	^76^
			*CEP135*	Milk production	^77^
			*AASDH*	Metabolism	^78^
Chr6:78796013–79165093	Chr6:79007752–79203136	0	*HOPX*	Sperm traits	^79^
Chr8:52949829–52997226	Chr8:52676280–52949829	0	*BACH2*	Immune system	^80^
Chr9:7615407–8155540	Chr9:7838010–7948027	0			
Chr10:19347073–19637375	Chr10:18607317–18670320	677	*RUBCNL*	Vertebrate autophagy	^81^
			*LRCH1*	Immune system	^82^
			*HTR2A*	Adaptive traits	^83^
Chr10:66683508–66747944	Chr10:65063529–65212062	977	*SLITRK6*	Milk production traits	^73^
Chr10:83028244–85695176	Chr10:85120520–85216381	0	*HS6ST3*	Growth traits	^84^
			*STK24*	Immune response	^85^
			*SLC15A1*	Development of growth	^75^
			*GPR18*	Immune system	^86^
			*TM9SF2*	Fertility	^87^
			*DOCK9*	Disease resistance	^88^
Chr12:27323634–27702125	Chr12:27961834–28032557	260	*DUSP10*	Immunity inflammation	^89^
Chr14:12743159–13111196	Chr14:13401157–13465959	290	*FOXC2*	Immune system	^90^
Chr15:75842476–75936079	Chr15:76144260–76212569	208			
Chr16:47568518–47871748	Chr16:46887875–47002471	566			
Chr17:23675899–23828049	Chr17:23538968–23738050	0			
Chr17:23675899–23828049	Chr17:23988299–24240466	0			
Chr21:18899984–19383548	Chr21:18655157–18820162	80	*LRFN4*	Ovarian cancer	^91^
Chr21:46501772–46700862	Chr21:46501772–46628225	0	*TENM4*	Nervous system development	^92^
			*NARS2*	Energy deficiency disorders	^93^
Chr:25:14234573:14644765	Chr25:14561562–14644765	0	*PHYHIPL*	Growth and wool traits	^94^
Chr:25:34269680:34689602	Chr25:34845271–34945170	156	*KCNMA1*	Growth traits	^95^
			*DLG5*	Muscle growth	^96^
			*POLR3A*	Immune response	^97^
			*RPS24*	Heat stress	^98^

The CNVRs identified in different breeds have previously compared in a variety of species including sheep^23^, cattle^99^ and chicken^100^ to evaluate the reliability of the detected regions consisting CNVs. The high proportion of overlapped CNVRs observed in the current study between Iranian and Afghanistan sheep breeds may probably reflect this fact that these breeds have been influenced by similar evolutionary events, mostly due to their close geographic distribution and may some gene flow occurred between these breeds in the past.

In general, this study is an important step towards the generation of a CNV map and provides an important resource for studies of ovine genomic variation in indigenous sheep of Afghanistan. However, the number of animals used in the current study may be one of the limitations affecting the number of CNVs identified in this study. Although, it should be considered that this is the only genomic information available from Afghan local sheep breeds and most of the similar published reports have been performed with almost the same or lower sample size in different indigenous sheep breeds^7,21,42,50,57,94,101^, however, there were validation or phenotypes in most of the previous studies. For example, quantitative PCR (qPCR) has been used to confirm subsets of CNVs identified in different studies^42,101^. Given that no validation of our findings has been performed in the current study, the results presented in this study is purely exploratory and external validation will be necessary to confirm the findings. It seems by increasing the number of genotyped samples in the future researches, we would be able to shed some new lights on the domestication event and the history of Afghan sheep. Also, further research on phenotypic associations would help understanding the role of CNVs in the formation of the local sheep breeds. Our results offer meaningful genomic insights that will help to guide future research and to provide a preliminary basis for the future exploration of the relationship between CNVs and economically important phenotypes of sheep.

## Conclusion

Overall, the first map of CNVs on the genome of Afghan sheep breeds was developed in this study using genome-wide information. A total number of 411 CNVs and 376 CNVRs were identified that cover 144 Mb of the genome. Since each CNV variant contains more length of the DNA, comparing to single nucleotide polymorphism (SNP) markers, and a variety of previously published studies suggest that these regions have been associated with economically important traits, it seems CNVs can provide better information for realizing the missing inheritance related to complex traits in Afghan sheep breeds. The results of this study showed that the genes reported in the genomic regions containing CNVRs in this study are associated with various metabolic pathways including reproductive traits, carcass characteristics, and body weight, immune and musculoskeletal system. The results of the present study provide valuable information for analyzing the association of CNVs with economically important phenotypes in Afghan sheep.

## Supplementary Information

Supplementary Table S1.

## Data Availability

All CNVRs identified in Afghan sheep breeds have been deposited in figshare repository (https://www.figshare.com) with accession no. 10.6084/m9.figshare.16577954.v1. The genomic information used in this study belongs to private sheep breeding programs, so we do not have authorization to share it and are available from the corresponding authors upon reasonable request.
